# Generalized Ordinal Patterns and the KS-Entropy

**DOI:** 10.3390/e23081097

**Published:** 2021-08-23

**Authors:** Tim Gutjahr, Karsten Keller

**Affiliations:** Institute of Mathematics, University of Lübeck, D-23562 Lübeck, Germany; gutjahr@math.uni-luebeck.de

**Keywords:** ordinal patterns, measure-preserving dynamical system, Kolmogorov–Sinai entropy, permutation entropy, ergodic theory

## Abstract

Ordinal patterns classifying real vectors according to the order relations between their components are an interesting basic concept for determining the complexity of a measure-preserving dynamical system. In particular, as shown by C. Bandt, G. Keller and B. Pompe, the permutation entropy based on the probability distributions of such patterns is equal to Kolmogorov–Sinai entropy in simple one-dimensional systems. The general reason for this is that, roughly speaking, the system of ordinal patterns obtained for a real-valued “measuring arrangement” has high potential for separating orbits. Starting from a slightly different approach of A. Antoniouk, K. Keller and S. Maksymenko, we discuss the generalizations of ordinal patterns providing enough separation to determine the Kolmogorov–Sinai entropy. For defining these generalized ordinal patterns, the idea is to substitute the basic binary relation ≤ on the real numbers by another binary relation. Generalizing the former results of I. Stolz and K. Keller, we establish conditions that the binary relation and the dynamical system have to fulfill so that the obtained generalized ordinal patterns can be used for estimating the Kolmogorov–Sinai entropy.

## 1. Introduction

In 2002, Bandt and Pompe introduced so-called permutation entropy [1]. This entropy has been established in non-linear dynamical system theory and time series analysis, including applications in many fields from biomedicine to econophysics (compare with Zanin et al. [2]). It is a crucial point that permutation entropy is theoretically justified by asymptotic results relating it to Kolmogorov–Sinai entropy (KS entropy, also called metric entropy) which is the central complexity measure for dynamical systems. The important relationship of permutation entropy and KS entropy was first observed and mathematically founded for piece-wise monotone dynamical systems by Bandt et al. [3].

The (empirical) concept of permutation entropy is based upon analyzing the distribution of ordinal patterns in a time series or the underlying system. In this paper, we concentrate on a *measure-preserving dynamical system* (Ω,A,μ,T), i.e., a probability space (Ω,A,μ) equipped with a measurable map T:Ω→Ω satisfying $\mu (T^{-1}\left(A\right))=\mu \left(A\right)$ for all A∈A.

Given a random variable X:Ω→R, in this paper, an *ordinal pattern of length*n∈N with respect to *X* is considered as a subset of the state space Ω. It is indicated by a permutation $\pi =(\pi_{0},\pi_{1},\ldots ,\pi_{n-1})$ of {0,1,…,n−1} and defined by

(1) $$P_{\pi }:=\left\{\omega \in \Omega |X\left(T^{\pi_{0}}\left(\omega \right)\right)\leq X\left(T^{\pi_{1}}\left(\omega \right)\right)\leq \ldots \leq X\left(T^{\pi_{n-1}}\left(\omega \right)\right)\right\}.$$

(Usually, ordinal patterns are defined in the range of *X*, i.e., for the vectors $\left(X\left(T\left(\omega \right)\right),X\left(T^{1}\left(\omega \right)\right),\ldots ,X\left(T^{n-1}\left(\omega \right)\right)\right)$). The collection of ordinal patterns:

$$O\;P_{}\left(n\right):=\left\{P_{\pi }|\pi \;\mathrm{is}\;a\;\mathrm{permutation}\;\mathrm{of}\;\mathrm{length}\;n\right\}$$

is a partition of Ω.

In the rest of this section, we assume that *X* preserves enough information about the given system in a certain sense. This is particularly the case if Ω is contained in R and *X* is the identity map. A precise general description of the assumption is given when presenting the results of this paper. It was shown in [4] that, under not too restrictive further conditions, the probability distribution on the partitions $O\;P_{}\left(n\right)$ for n∈N can be used for determining the KS entropy of the given system. The reason is that, roughly speaking, under these conditions, $O\;P_{}\left(n\right)$ is able to separate the orbits of the system if n→∞.

In order to address the problem that this paper is concerned with, we give a description of ordinal patterns being slightly different from the above. One can determine to which ordinal pattern $P_{\pi }$ of length *n* a point ω belongs to if, for all (s,t) in:

(2) $$E_{n}=\left\{\left(s,t\right)\in N_{0}^{2}|0\leq s<t\leq n-1\right\},$$

one knows whether $X\left(T^{s}\left(\omega \right)\right)\leq X\left(T^{t}\left(\omega \right)\right)$ holds true or not. In other words, there exists a set $A\subseteq E_{n}$ such that:

(3) $$\begin{array}{cc} P_{\pi }= & \underset{(s,t)\in A}{⋂}\left\{\omega \in \Omega |\left(X\left(T^{s}\left(\omega \right)\right),X\left(T^{t}\left(\omega \right)\right)\right)\in R\right\}\\ \cap & \underset{\left(s,t\right)\in E_{n}\backslash A}{⋂}\left\{\omega \in \Omega |\left(X\left(T^{s}\left(\omega \right)\right),X\left(T^{t}\left(\omega \right)\right)\right)\in R^{2}\backslash R\right\}, \end{array}$$

where:

(4) $$R:=\{\left(x,y\right)\in R^{2}|x>y\}.$$

The above set contains all the points ω∈Ω that satisfy $X\left(T^{s}\left(\omega \right)\right)>X\left(T^{t}\left(\omega \right)\right)$ for (s,t)∈A and $X\left(T^{s}\left(\omega \right)\right)\leq X\left(T^{t}\left(\omega \right)\right)$ for $\left(s,t\right)\in E_{n}\backslash A$. Note that, given some arbitrary $A\subseteq E_{n}$, the set on the right hand side of (3) can be empty. In the case that it is non-empty, it coincides with some ordinal pattern $P_{\pi }$ of length *n*.

While Equation (3) might be a bit more abstract than (1), it shows a way to generalize the concept of ordinal patterns on the basis of replacing the set *R* in (4) by some arbitrary Borel subset *R* of $R^{2}$, also to investigate why ordinal patterns are so successful.

**Definition** **1.** *We call a non-empty Borel subset R of $R^{2}$ discriminating relation.*

**Figure 1:**
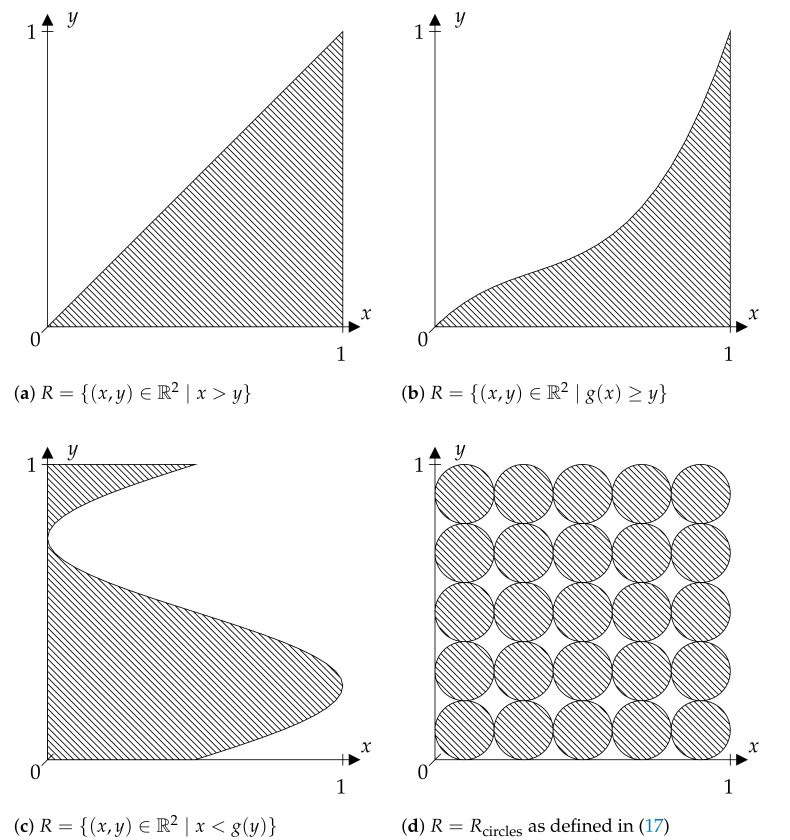
This figure illustrates some special discriminating relations *R* (striped areas) considered in Section 4. Only the part of *R* contained in [0, 1[^2^ is shown (compare the corresponding remarks in Section 4).

The figures given in this paper show different discriminating relations *R*. In each case, only the part of *R* contained in ${[0,1[}^{2}$ is presented. Note that in the case that *X* maps Ω into [0,1[, the restriction of *R* itself to this part would not change anything. Figure 1a illustrates *R* as given in (4), again only on ${[0,1[}^{2}$. In the case of such an *R*, note that tan(π(X−1/2)) mapping [0,1[ into [−∞,∞[ would not make a difference to a given *X* for our considerations, since order relations and associated partitions are preserved.

Given some discriminating relation, *generalized ordinal patterns of length* *n* with respect to *X* are given as the non-empty sets defined by the right hand side of (3) for some $A\subseteq E_{n}$. Obviously, they also form a partition of Ω. The question that arises is what a discriminating relation *R* should look like, such that those generalized ordinal patterns inhibit the same nice properties the original ordinal patterns had. More precisely, we ask the following question:

**Main Question**. *Under what conditions on a discriminating relation R the partitions given by the generalized ordinal patterns determine the KS entropy of a dynamical system?*

Why is this determination of entropy, which is precisely described by formula (10) in Theorem 1 interesting? For answering this question, interpret *X* as an observable, ω as the initial state of the given system and $X\left(\omega \right),X\left(T\left(\omega \right)\right),X\left(T^{2}\left(\omega \right)\right),X\left(T^{3}\left(\omega \right)\right),\ldots$ as the measured values at times 0,1,2,3,…. Determining (generalized) ordinal patterns on the basis of those values is a symbolization, where a symbol obtained is the (generalized) ordinal pattern containing ω. Generally, symbolization means a coarse-graining of the state space underlying a system, where each point is assigned one of finitely many given symbols. Instead of considering the precise development of the system, one is interested in the change of symbols in the course of time, justifying the naming of the method symbolic dynamics. Note that a symbolization is equivalent to partitioning the state space into classes of states (with the same symbol).

The reason for obtaining the full entropy from the (generalized) ordinal patterns is, roughly speaking, that the symbol system obtained has high potential for separating orbits. Such kinds of successful symbolizations are important, for example, in big data analysis, see, e.g., Smith et al. [5].

The above question was first considered in [6], where the authors basically showed that sets of the form:

$$R=\{\left(x,y\right)\in R^{2}|g\left(x\right)\geq y\}$$

lead to generalized ordinal patterns that, under some conditions, can be used to determine the entropy if g:R→R is measurable and one-to-one. Such an *R* is shown in Figure 1b and will be discussed in Section 4 as well as another *R* illustrated in Figure 1.

In this paper, we consider general sets $R\subseteq R^{2}$ that cannot necessarily be described by functions and inequalities and establish some conditions under which the entropy can be determined using those sets. As in [6], the discussion also includes a generalization of the sets $E_{n}$ given by (2) and is conducted in a multidimensional framework. In particular, the results give insights as to why the basic ordinal approach and generalizations are working.

It is instructive to discuss the partition of $R^{2}$ into *R* and $R^{2}\backslash R$ from the viewpoint of symbolic dynamics. In contrast to classical symbolization approaches with symbolizing only in the range of single “measurements” *x*, the symbolization of pairs (x,y) via the partition $\left\{R,R^{2}\backslash R\right\}$ also regards some kind of link between *x* and *y* if *R* lies “diagonal” in a certain sense. We will discuss this constellation, which explains the success of ordinal patterns in a wider context, more precisely in Section 5.

**Figure 2:**
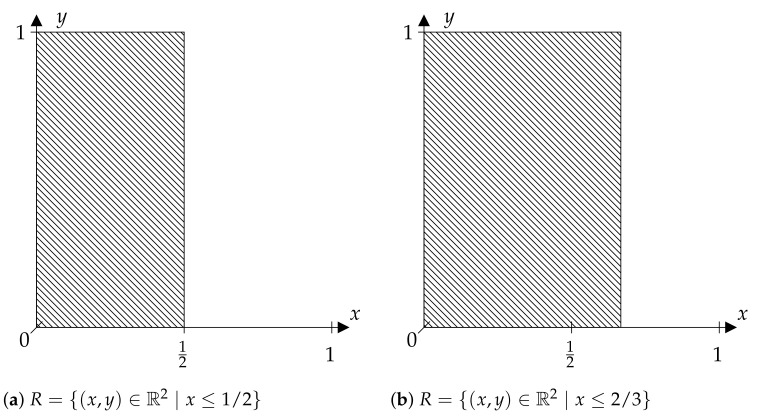
“Non-diagonal” discriminating relations.

A completely different constellation is given for the sets *R* shown in Figure 2. Here, *R* is obtained as a half-plane from a “horizontal” division of $R^{2}$. If, for example, Ω=[0,1[, A is the Borel σ-algebra and μ the Lebesgue measure on Ω, and if *T* is the tent map on Ω, meaning that:

(5) $$T\left(\omega \right)=\left\{\begin{array}{cc} 2\omega & \;\mathrm{if}\;0\leq \omega <\frac{1}{2},\\ 2-2\omega & \;\mathrm{if}\;\frac{1}{2}\leq \omega <1, \end{array}\right.$$

and *X* is the identity map, then the location of the horizontal cut is substantial.

On the one hand, $R=\{\left(x,y\right)\in R^{2}|x\leq 2/3\}$ (Figure 2b) does not discriminate enough to obtain the KS entropy of the given system, and on the other hand, there is enough discrimination by $R=\{\left(x,y\right)\in R^{2}|x\leq 1/2\}$ (Figure 2a) due to the fact that $\{[0,\frac{1}{2}[,[\frac{1}{2},1\left[\right\}$ is a generating partition for *T*. In the situation considered, there is no additional information given by the measurements (x,y) relative to measurement *x*, hence *R* provides nothing more than a classical symbolization. For a detailed discussion of these facts, see [6].

The rest of this paper is organized as follows. Section 2 provides the notions and concepts being necessary for formulating the main statement of this paper in Section 3. This statement is rather abstract and general and has to be considered in relation to some special cases discussed in Section 4 and making our ideas and findings transparent. Section 5 is devoted to the proof of our main statement.

## 2. Preliminaries

Throughout this paper, (Ω,A,μ,T) will be a measure-preserving dynamical system.

### 2.1. Some Notions

We will write B=B(R) or $B(R^{d})$ for the Borel σ-algebra on R or $R^{d};d\in N$, respectively. Given a random variable X:Ω→R, by $\mu_{X}$ we denote the push-forward measure of μ with regard to *X*, i.e., $\mu_{X}\left(A\right):=\mu \left(X^{-1}\left(A\right)\right)$ for all A∈B(R). The measure $\mu_{X}\times \mu_{X}=\mu_{X}^{2}$ is the product measure, i.e., $\mu_{X}^{2}\left(A\times B\right)=\mu_{X}\left(A\right)\mu_{X}\left(B\right)$ for all A,B∈B(R).

For some Borel set $R\in B(R^{2})$, we define the function $f_{X}^{R}:R\to \left[0,1\right]$ by

(6) $$f_{X}^{R}\left(x\right):=\mu \left(\left\{\omega \in \Omega |\left(x,X\left(\omega \right)\right)\in R\right\}\right).$$

If it is clear from the context which set $R\in B(R^{2})$ is considered, we simply write $f_{X}$ instead of $f_{X}^{R}$. The function $f_{X}$ can be represented as the integral:

$$f_{X}\left(x\right)=\int 1_{R}\left(x,y\right)\;d\mu_{X}\left(y\right).$$

Since $1_{R}$ is integrable with regard to $\mu_{X}^{2}$, $f_{X}$ is integrable and therefore, also measurable by Fubini’s Theorem.

The complement $R^{2}\backslash R$ of a set $R\subseteq R^{2}$ will be denoted by $R^{c}$. The notation ∂R will be used for the boundary of a set *R*, i.e., the closure of *R* without its interior.

### 2.2. Entropy

The *Shannon entropy* of a finite partition P⊂A of Ω is defined as

$$H\left(P\right):=-\sum_{P\in P}\mu \left(P\right)\log\left(\mu \left(P\right)\right).$$

The refinement of two partitions P,Q⊂A of Ω is given by

P∨Q:={P∩Q≠∅∣P∈P,Q∈Q}.

For a finite collection of partitions $P_{i}\subset A,i\in \left\{1,2,\ldots ,n\right\}$ of Ω, one analogously defines:

$$\overset{n}{\underset{i=1}{⋁}}P_{i}:=\left\{\overset{n}{\underset{i=1}{⋂}}P_{i}\neq \emptyset |P_{i}\in P_{i}\;\mathrm{for}\;\mathrm{all}\;i\in \left\{1,2,\ldots ,n\right\}\right\}.$$

The *entropy rate* of a finite partition P⊂A of Ω is defined as

$$h\left(T,P\right):=\lim_{n\to \infty }\frac{1}{n}H\left(\overset{n-1}{\underset{t=0}{⋁}}T^{-t}\left(P\right)\right),$$

where $T^{-t}\left(P\right)=\left\{T^{-t}\left(P\right)|P\in P\right\}$. For the existence of the limit in the formula, see, e.g., [7]. We are interested in determining the *Kolmogorov–Sinai entropy* of a system, which is defined as

$$h\left(T\right):=\underset{P}{\sup}h\left(T,P\right),$$

where the supremum is taken over all finite partitions of Ω in A.

Note that the Kolmogorov–Sinai entropy serves as the central complexity measure for dynamical systems and can be considered as a reference for other complexity measures, including in data analysis. Roughly speaking, it measures the mean information obtained by each iteration step. Since the Kolmogorov–Sinai entropy is the supremum of the entropy rates of all finite partitions, its determination and its estimation in a practical context are not easy and it is of some interest to find natural finite partitions for which the entropy rate is near the Kolmogorov–Sinai entropy. This is also a motivation for considering ordinal patterns and its generalization in this paper.

### 2.3. σ-Algebras

Given a family of sets $A_{i}\in A,i\in I$, by $\sigma (A_{i}|i\in I)$ we denote the smallest σ-algebra containing all sets $A_{i}$. Analogously, for a family of partitions $P_{i}\subset A,i\in I$ of Ω, we define $\sigma (P_{i}|i\in I)$ as the smallest σ-algebra containing all partitions $P_{i}$ as subsets. Given *d*-dimensional random vectors $\left(Y_{i,1},Y_{i,2},\ldots ,Y_{i,d}\right):\Omega \to R^{d},i\in I$, we define:

$$\sigma \left(\left(Y_{i,1},Y_{i,2},\ldots ,Y_{i,d}\right)|i\in I\right):=\sigma \left({\left(Y_{i,1},Y_{i,2},\ldots ,Y_{i,d}\right)}^{-1}\left(B\right)|i\in I,B\in B\left(R^{d}\right)\right)$$

as the smallest σ-algebra containing all preimages of Borel sets. When comparing two σ-algebras $A_{1},A_{2}\subseteq A$, we ignore sets with measure 0, i.e., we write:

$$A_{1}\subseteq_{\mu }A_{2}$$

if for all $A_{1}\in A_{1}$ there exists an $A_{2}\in A_{2}$ with $\mu (\left(A_{1}\backslash A_{2}\right)\cup \left(A_{2}\backslash A_{1}\right))=0$. In this sense, $A_{1}{=}_{\mu }A_{2}$ means that $A_{1}\subseteq_{\mu }A_{2}$ and $A_{2}\subseteq_{\mu }A_{1}$.

## 3. The Main Statement

Recall that a measure-preserving dynamical system (Ω,A,μ,T) is *ergodic* if for each B∈A with $T^{-1}\left(B\right)=B$ it holds μ(B)∈{0,1}, meaning that the system does not divide into proper parts.

Referring to Section 1, we give some preparation for stating our main result. Recall that for defining (generalized) ordinal patterns it was basic to know whether $(X\left(T^{s}\left(\omega \right)\right),X\left(T^{s}\left(\omega \right)\right))\in R$ or $\left(X\left(T^{s}\left(\omega \right)\right),X\left(T^{s}\left(\omega \right)\right)\right)\in R^{2}\backslash R$ for ω∈Ω and a random variable *X* on Ω, where “time” pairs (s,t) were taken from the sets $E_{n}$ (see (2)). In order to also allow reducing the number of necessary “comparisons”, we relax the definition of the sets $E_{n}$ leading to the following concept.

**Definition** **2.** *We call a sequence ${\left(E_{n}\right)}_{n\in N}$ with $E_{1}\subseteq E_{2}\subseteq E_{3}\subseteq \ldots \subseteq N_{0}^{2}$* timing*, if $E_{n}$ contains finitely many elements and if there exists a sequence ${\left(a_{n}\right)}_{n\in N}\in N_{0}^{N}$ with:*

(7) $$\underset{N\to \infty }{lim sup}\frac{\#\left\{n\in \left\{1,2,\ldots ,N\right\}|\left(a_{n},a_{n}+n\right)\in {⋃}_{k=1}^{\infty }E_{k}\right\}}{N}=1.$$

Formula (7) roughly says that nearly each “temporal” distance is available for “comparisons”. It guarantees that enough time-pairs are considered to not have any loss of information contained in the “thinned out” generalized ordinal patterns relative to the “full” generalized ordinal patterns.

**Remark** **1.** *In the first paper on generalized ordinal patterns ([6]), a timing ${\left(E_{n}\right)}_{n\in N}$ was differently defined: the authors of that paper called a sequence of finite sets $E_{1}\subseteq E_{2}\subseteq E_{3}\subseteq \ldots \subseteq N_{0}^{2}$ timing, if there exists an increasing sequence ${\left(a_{n}\right)}_{n\in N}$ such that for all n∈N:*

(i) *$E_{n}\subseteq {\left\{a_{0},a_{1},\ldots ,a_{n}\right\}}^{2}$,*
(ii) *for all $s\in \{a_{0},a_{1},\ldots ,a_{n}\}$, there exists a $t\in \{a_{0},a_{1},\ldots ,a_{n}\}$ with s≠t and $\left(s,t\right)\in E_{n}$,*
(iii) *${\left(id,T^{n}\right)}^{-1}\left(R_{i}\right)\subseteq_{\mu }\sigma \left({⋁}_{\left(s,t\right)\in E_{k}}{\left(T^{s},T^{t}\right)}^{-1}\left(R_{i}\right)|k\in N\right)$ for all i∈{1,2,…,d}*

*hold true. Note that the last condition does not only depend on the timing ${\left(E_{n}\right)}_{n\in N}$ but also on T and $X=(X_{1},X_{2},\ldots ,X_{d})$. Instead of those three conditions, we instead simply require that almost all differences can be found in the timing.*

Given a random vector $\left(X_{1},X_{2},\ldots ,X_{d}\right)$ and $R\in B(R^{2})$, we define the partition:

$$R_{i}:={\left(X_{i},X_{i}\right)}^{-1}\left(\left\{R,R^{c}\right\}\right)\;=\{{\left(X_{i},X_{i}\right)}^{-1}\left(R\right),{\left(X_{i},X_{i}\right)}^{-1}\left(R^{c}\right)\}$$

for all i∈{1,2,…,d}, which is equal to:

$$\begin{array}{cc} R_{i}=\{ & \{\left(\omega_{1},\omega_{2}\right)\in \Omega^{2}|\left(X_{i}\left(\omega_{1}\right),X_{i}\left(\omega_{2}\right)\right)\in R\},\\ \; & \{\left(\omega_{1},\omega_{2}\right)\in \Omega^{2}|\left(X_{i}\left(\omega_{1}\right),X_{i}\left(\omega_{2}\right)\right)\notin R\}\}. \end{array}$$

Then, for $E_{n}$ as given in (2) the partition ${⋁}_{\left(s,t\right)\in E_{k}}{\left(T^{s},T^{t}\right)}^{-1}\left(R_{i}\right)$ is no more than the partition of generalized ordinal patterns with respect to $X_{i}$ defined in Section 1 and ${⋁}_{i=1}^{d}{⋁}_{\left(s,t\right)\in E_{k}}{\left(T^{s},T^{t}\right)}^{-1}\left(R_{i}\right)$ can be considered as the partition of generalized ordinal patterns with respect to $\left(X_{1},X_{2},\ldots ,X_{d}\right)$.

The proof of the following main theorem of the paper is given in Section 5.

**Theorem** **1.** *Let (Ω,A,μ,T) be an ergodic measure-preserving dynamical system, $X=\left(X_{1},X_{2},\ldots ,X_{d}\right):\Omega \to R^{d}$ be a random vector, $R\in B(R^{2})$ be a discriminating relation and ${\left(E_{n}\right)}_{n\in N}$ be a timing. Assume that the following conditions are valid:*

(8) $$\begin{array}{c} There\;exists\;a\;countable\;set\;C\subseteq R^{2}with:\;\\ \;\mu_{X_{i}}^{2}\left(\partial R\backslash C\right)=0\;for\;all\;i\in \left\{1,2,\ldots ,d\right\}.\;\; \end{array}$$

(9) $$\begin{array}{c} There\;exists\;a\;random\;variable\;Y:\Omega \to Rwith:\;\\ \#Y\left(\Omega \right)<\infty \;and\;A{=}_{\mu }\sigma \left(\left(f_{X_{i}}^{R}\circ X_{i}\circ T^{t},Y\right)|t\in N_{0},i\in \left\{1,2,\ldots ,d\right\}\right). \end{array}$$

*Then:*

(10) $$h\left(T\right)=\lim_{k\to \infty }h\left(T,\overset{d}{\underset{i=1}{⋁}}\underset{\left(s,t\right)\in E_{k}}{⋁}{\left(T^{s},T^{t}\right)}^{-1}\left(R_{i}\right)\right).$$

*holds true.*

At first glance, conditions (8) and (9), being sufficient for (10), are looking very special. The considerations in the following section will, however, elucidate their role and show that they are relatively general. Roughly speaking, (8) says that the distribution of pairs of “independent measurements” with respect to $X_{i}$ is discrete on the boundary of *R*. Condition (9) is an orbit separation condition based on the involved “measurements” and the functions $f_{X_{i}}^{R}$. In general:

$$A{⊇}_{\mu }\sigma \left(\left(f_{X_{i}}^{R}\circ X_{i}\circ T^{t},Y\right)|t\in N_{0},i\in \left\{1,2,\ldots ,d\right\}\right)$$

holds true because all functions involved in (9) are A measurable. Therefore, (9) is equivalent to

$$A\subseteq_{\mu }\sigma \left(\left(f_{X_{i}}^{R}\circ X_{i}\circ T^{t},Y\right)|t\in N_{0},i\in \left\{1,2,\ldots ,d\right\}\right)$$

The inclusion of the random variable *Y* provides some further separation and allows the above inclusion to hold true for a wider class of dynamical systems than, for example, the ones considered in [6]. In the case that *Y* is constant, it can also be omitted. In theory, *Y* should be chosen to take different values on those sets on which $f_{X_{i}}^{R}\circ X_{i}\circ T^{t}$ takes the same values for i∈{1,2,…,d} and $t\in N_{0}$. In practice, the fact that such a random variable *Y* exists is sufficient and *Y* does not need to be explicitly specified. An example is given in Section 4.5.

## 4. Special Cases

In the following, we discuss some special situations where the assumptions of Theorem 1, i.e., (8) and (9), are satisfied. Lemma 2 provides an easy-to-check condition, that of when (8) holds true. It is more difficult to see, when the condition (9) is satisfied. Roughly speaking, this condition is fulfilled if $X=(X_{1},X_{2},\ldots ,X_{d})$ together with *Y* can uniquely describe the outcomes of the whole dynamical system and applying $f_{X_{i}}$ to the results of X is, in some sense, “reversible” for all i∈{1,2,…,d}. In other words, $X=(X_{1},X_{2},\ldots ,X_{d})$ together with *Y* preserve the information of the system and there is no information loss for the symbolization. The first means that:

$$A{=}_{\mu }\sigma \left(\left(X_{i}\circ T^{t},Y\right)|t\in N_{0},i\in \left\{1,2,\ldots ,d\right\}\right),$$

which obviously follows from

$$A{=}_{\mu }\sigma \left(\left(f_{X_{i}}^{R}\circ X_{i}\circ T^{t},Y\right)|t\in N_{0},i\in \left\{1,2,\ldots ,d\right\}\right).$$

To describe the range of outcomes of the random variables *X* on a probability space (Ω,A,μ), we will use its cumulative distribution functions $F_{X}:R\to \left[0,1\right]$ defined by

$$F_{X}\left(x\right):=\mu_{X}\left(\right]-\infty ,x]).$$

When applying the cumulative distribution functions $F_{X}$ to the outcomes *X* of a system, we do not lose any essential information about the system, according to the following lemma. This lemma is a simple modification of Lemma A.3 in [6].

**Lemma** **1.** *Let (Ω,A,μ) be a probability space, X:Ω→R be a random variable and g:R→R be a B−B measurable function which maps Borel sets to Borel sets and satisfies the following property:*

(11) $$For\;all\;x\in R\;it\;holds\;\mu (X^{-1}\left(g^{-1}\left(g\left(\right]-\infty ,x\right]\right))\;\backslash \;]-\infty ,x]))=0$$

*Then, $\sigma \left(g\circ X\right){=}_{\mu }\sigma \left(X\right)$. In particular, $\sigma \left(g\circ X\right){=}_{\mu }\sigma \left(X\right)$ if $g=F_{X}$ or g is injective on X(Ω).*

Condition (11) in the above Lemma is a slightly weaker condition on *g* than injectivity. If *g* is injective, then $g^{-1}\left(g\left(\right]-\infty ,x\right]))=]-\infty ,x]$ will hold true for all x∈R and condition (11) will be satisfied. More general, condition (11) can still be true if *g* is not necessarily injective but if all sets on which *g* is not injective, which are given by $X^{-1}\left(g^{-1}\left(g\left(\right]-\infty ,x\right]\right))\;\backslash \;]-\infty ,x])$ for all x∈R, have measure 0. For example, this is true if *g* is equal to the cumulative distribution function.

### 4.1. On the Boundary of *R*

The condition (8) in Theorem 1, that the boundary of *R* apart from countably many points has measure 0, holds true for all “simple” sets *R*. In the following lemma, we specify what we mean by “simple”.

**Lemma** **2.** *Let (Ω,A,μ) be a probability space, $\left(X_{1},X_{2},\ldots ,X_{d}\right)$ be a random vector and $R\in B(R^{2})$. If, for all i∈{1,2,…,d}:*

$$\begin{array}{cc} \; & \partial R\cap \left(\left\{x\right\}\times R\right)\;\mathrm{is}\;\mathrm{countable}\;\mathrm{for}\;\mu_{X_{i}}\text{-}\mathrm{almost}\;\mathrm{all}\;x\in R\\ \mathrm{or}\;\; & \partial R\cap \left(R\times \left\{y\right\}\right)\;\mathrm{is}\;\mathrm{countable}\;\mathrm{for}\;\mu_{X_{i}}\text{-}\mathrm{almost}\;\mathrm{all}\;y\in R, \end{array}$$

*then R satisfies (8), i.e., there exists a countable set $C\subseteq R^{2}$ with:*

$$\mu_{X_{i}}^{2}\left(\partial R\backslash C\right)=0\;\;\mathrm{for}\;\mathrm{all}\;i\in \left\{1,2,\ldots ,d\right\}.\;$$

**Proof.**  Consider the sets:

$$A_{i}:=\left\{x\in R|\mu_{X_{i}}\left(\left\{x\right\}\right)>0\right\}$$

for i∈{1,2,…,d}, which, obviously, are countable. Set:

$$C:=\overset{d}{\underset{i=1}{⋃}}A_{i}\times A_{i}.$$

Let i∈{1,2,…,d}. If {y∈R∣(x,y)∈∂R} is countable for $\mu_{X_{i}}$-almost all x∈R, Fubini’s theorem implies:

$$\begin{array}{cc} \mu_{X_{i}}^{2}\left(\partial R\backslash C\right) & =\int \int 1_{\partial R\backslash C}\left(x,y\right)\;d\mu_{X_{i}}\left(y\right)\;d\mu_{X_{i}}\left(x\right)\\ \; & =\int \mu_{X_{i}}\left(\left\{y\in R|\left(x,y\right)\in \partial R\backslash C\right\}\right)\;d\mu_{X_{i}}\left(x\right)\\ \; & =\int \sum_{\begin{array}{c} y\in R:\\ (x,y)\in \partial R\backslash C \end{array}}\mu_{X_{i}}\left(\left\{y\right\}\right)\;d\mu_{X_{i}}\left(x\right)\\ \; & =\int 0\;d\mu_{X_{i}}\left(x\right)=0. \end{array}$$

Analogously, one can show the same if {x∈R∣(x,y)∈∂R} is countable for $\mu_{X_{i}}$-almost all y∈R. □

**Remark** **2.** *The patterns visualized in Figure 1 could also be defined on the whole real axis instead of a bounded interval by, for example, applying the transformation φ(x)=tan(π(x−1/2)). Then, $\tilde{R}=\left(\varphi \times \varphi \right)\left(R\right)$ is a pattern defined on $R^{2}$ if R is defined on ${[0,1[}^{2}$.*

In the following three subsections, *Y* is assumed to be constant, and hence can be omitted.

### 4.2. Basic Ordinal Patterns

If:

$$R=\{\left(x,y\right)\in R^{2}|x\geq y\}$$

(see Figure 1a), then $f_{X_{i}}^{R}$ is just the distribution function of $\mu_{X_{i}}$, i.e., $f_{X_{i}}^{R}=F_{X_{i}}$. Since ∂R∩{x}×R={(x,x)} is finite for all x∈R, (8) holds true by Lemma 2. According to Lemma 1, one has:

(12) $$\sigma \left(X_{i}\circ T^{t}\right)\subseteq_{\mu }\sigma \left(F_{X_{i}}\circ X_{i}\circ T^{t}\right)$$

for all $t\in N_{0}$ and i=1,2,…,n. Therefore:

(13) $$\begin{array}{cc} A & {=}_{\mu }\sigma \left(\left(f_{X_{i}}^{R}\circ X_{i}\circ T^{t}\right)|t\in N_{0},i\in \left\{1,2,\ldots ,d\right\}\right)\\ \; & =\;\sigma \left(\left(F_{X_{i}}\circ X_{i}\circ T^{t}\right)|t\in N_{0},i\in \left\{1,2,\ldots ,d\right\}\right) \end{array}$$

is equivalent to:

(14) $$A{=}_{\mu }\sigma \left(\left(X_{i}\circ T^{t}\right)|t\in N_{0},i\in \left\{1,2,\ldots ,d\right\}\right).$$

By Theorem 1, for ergodic systems, condition (14) implies (10). A more general statement also includes a large class of non-ergodic systems which was shown in [4]. Condition (14) is, for example, satisfied if $\Omega \in B(R^{d})$ and $X_{i}$ is the projection on the *i*-th coordinate for all i∈{1,2,…,d}, or if Ω is a compact Hausdorff space and $X=(X_{1},X_{2},\ldots ,X_{d})$ is injective and continuous. One can also use Taken’s theorem to argue that the set of maps $X:\Omega \to R^{d}$ that satisfy (14) is large in a certain topological sense. For both, see Keller [8].

### 4.3. Patterns Defined by “Injective” Functions

Let $X=(X_{1},X_{2},\ldots ,X_{d})$ be a random vector and consider now:

(15) R={(x,y)∈R∣g(x)≥y}

for a B−B measurable function g:R→R (see Figure 1b). Since ∂R∩({x}×R)={(x,g(x))} is finite for all x∈R, (8) holds true by Lemma 2. Moreover, one easily sees that $f_{X_{i}}^{R}=F_{X_{i}}\circ g$.

Now, suppose that:

(16) $$\sigma \left(F_{X_{i}}\right)\subseteq \sigma \left(F_{X_{i}}\circ g\right)$$

holds true for all i∈{1,2,…,d}. This directly yields:

$$\sigma \left(F_{X_{i}}\circ X_{i}\circ T^{t}\right)\subseteq \sigma \left(F_{X_{i}}\circ g\circ X_{i}\circ T^{t}\right)$$

for all i∈{1,2,…,d} and t∈N. Remember that $\sigma \left(X_{i}\circ T^{t}\right)\subseteq_{\mu }\sigma \left(F_{X_{i}}\circ X_{i}\circ T^{t}\right)$ holds true according to Lemma 1. Thus, (14) and (16) imply (10). When considering basic ordinal patterns in Section 4.2, we stated some conditions under which (14) holds true. It remains to consider when (16) is satisfied.

Assume that *g* maps Borel sets to Borel sets and is injective. This implies:

$$\sigma \left(g\circ X_{i}\circ T^{t}\right)=\sigma \left(X_{i}\circ T^{t}\right)$$

for all $t\in N_{0}$ and i∈{1,2,…,d}. Now, suppose that:

$$A=\sigma \left(F_{X_{i}}\circ X_{i}\circ T^{t}|t\in N_{0},i\in \left\{1,2,\ldots ,d\right\}\right).$$

holds true. This would then imply (16). However, the above equation only holds true μ-almost surely (see (13)). This can be a problem when applying the function *g* because there could exist sets B∈B with $\mu_{X_{i}}\left(B\right)=0$ but $\mu_{X_{i}}\left(g\left(B\right)\right)>0$. Additionally, we therefore need to require that $\mu_{X_{i}}\left(g^{-1}\left(B\right)\right)=0$ implies $\mu_{X_{i}}\left(B\right)=0$ for all B∈B.

Theorem 1 then provides the following statement:

**Corollary** **1.** *Let (Ω,A,μ,T) be an ergodic measure-preserving dynamical system, $X=\left(X_{1},X_{2},\ldots ,X_{d}\right):\Omega \to R^{d}$ be a random vector and ${\left(E_{n}\right)}_{n\in N}$ be a timing. Let further g:R→R be a B−B measurable function which maps Borel sets to Borel sets, is injective on $X_{i}\left(\Omega \right)$ and satisfies $\mu_{X_{i}}\left(g^{-1}\left(B\right)\right)=0\Rightarrow \mu_{X_{i}}\left(B\right)=0$ for all B∈B and i∈{1,2,…,d}. Let $R=\{\left(x,y\right)\in R^{2}|g\left(x\right)\geq y\}$.*

*Then, (14) implies (10). Moreover, (10) holds true if $\Omega \in B(R^{d})$ and $X_{i}$ is the projection on the i-th coordinate for all i∈{1,2,…,d} or if Ω is a compact Hausdorff space and $X=(X_{1},X_{2},\ldots ,X_{d})$ is injective and continuous.*

Note that the statements in Corollary 1, in principle, were shown in [6]. The case of basic ordinal patterns is included by g(x)=x for all x∈R.

### 4.4. Patterns Defined by “Surjective” Functions

Swapping coordinates in (15) yields the set:

R={(x,y)∈R∣x<g(y)}

(see Figure 1c) with (8) following from Lemma 2 and with:

$$\begin{array}{cc} f_{X_{i}}^{R}\left(x\right) & =\mu \left(\left\{\omega \in \Omega |\left(x,X_{i}\left(\omega \right)\right)\in R\right\}\right)=\mu \left(\left\{\omega \in \Omega |x<g\left(X_{i}\left(\omega \right)\right)\right\}\right)\\ \; & =\mu \left(\left\{\omega \in \Omega |g\left(X_{i}\left(\omega \right)\right)\in \;\right]x,\infty \left[\right\}\right)=1-F_{g\circ X_{i}}\left(x\right). \end{array}$$

**Corollary** **2.** *Let (Ω,A,μ,T) be an ergodic measure-preserving dynamical system, $X=\left(X_{1},X_{2},\ldots ,X_{d}\right):\Omega \to R^{d}$ be a random vector and ${\left(E_{n}\right)}_{n\in N}$ be a timing. Let further g:R→R be a B−B measurable function and let $R=\{\left(x,y\right)\in R^{2}|x<g\left(y\right)\}$. Then, the following holds:*

(i) *If $F_{g\circ X_{i}}$ is injective on $X_{i}\left(\Omega \right)$ for i∈{1,2,…,d}, (14) implies (10).*
(ii) *If $\Omega \in B(R^{d})$ and $X_{i}$ is the projection on the i-th coordinate for all i∈{1,2,…,d} or if Ω is a compact Hausdorff space and $X=(X_{1},X_{2},\ldots ,X_{d})$ is injective and continuous, if further μ(U)>0 for every non-empty open set U⊆Ω, g is continuous and $X_{i}\left(\Omega \right)\subseteq g\left(X_{i}\left(\Omega \right)\right)$, then (10) is valid in each of the following two cases:*
  (1) *For each i∈{1,2,…,d} and all $x_{1},x_{2}\in X_{i}\left(\Omega \right)$ with $x_{1}<x_{2}$, there exists some $y\in X_{i}\left(\Omega \right)$ with $x_{1}<y<x_{2}$,*
  (2) *Ω is connected.*

**Proof**. (i): If the above assumptions are satisfied and $F_{g\circ X_{i}}$ is injective on $X_{i}\left(\Omega \right)$ for all i∈{1,2,…,d}, then by Lemma 1 it holds that $\sigma \left(X_{i}\circ T^{t}\right)\subseteq_{\mu }\sigma \left(F_{g\circ X_{i}}\circ X_{i}\circ T^{t}\right)$ for all $t\in N_{0}$ and i∈{1,2,…,d}. This implies (9), hence, by Theorem 1 the statement (10).

(ii): Given the assumptions of (ii), we have to show that $F_{g\circ X_{i}}$ is injective on $X_{i}\left(\Omega \right)$ for all i∈{1,2,…,d}. If Ω is connected, then (1) is obviously satisfied. We can thus start from (1). Take $x_{1},x_{2}\in X_{i}\left(\Omega \right)$ with $x_{1}<x_{2}$. Then, $g^{-1}\left(\right]x_{1},x_{2}\left[\right)$ is non-empty and because $g\circ X_{i}$ is continuous, $X_{i}^{-1}\left(g^{-1}\left(\right]x_{1},x_{2}\right]))$ contains a non-empty open set. This implies that $F_{g\circ X_{i}}\left(x_{1}\right)<F_{g\circ X_{i}}\left(x_{2}\right)$. because every non-empty open set was assumed to have a strictly positive measure. □

Notice that, unlike in (15), it is not necessary that *g* is one-to-one.

### 4.5. Piecewise Patterns

The previous subsection illustrates that (9) is fulfilled if, roughly speaking, $\left(X_{1},X_{2},\ldots ,X_{d}\right)$ preserves all information and if $f_{X_{i}}^{R}$ is a $\mu_{X_{i}}$ almost surely invertible function for all i∈{1,2,…,d}. The finite-valued random variable *Y* in (9) can be used to weaken the condition of invertibility in the sense that only piecewise invertibility is needed where the different pieces are induced by the random variable *Y*.

For Ω=[0,1[ and an absolutely continuous measure μ, one could, for example, consider:

(17) $$R_{\mathrm{circles}}=\left\{\left(x,y\right)\in \Omega^{2}|{∥\left(kx\;\mathrm{mod}\;1,ky\;\mathrm{mod}\;1\right)-\left(0.5,0.5\right)∥}_{2}\leq 0.5\right\}$$

for any k∈N, as shown for k=5 in Figure 1d. The set *R* satisfies condition (9) with Y(ω)=i for ω∈[(i−1)/(2k),i/(2k)[ and i∈{1,2,…,2k}. The set *R* is a pattern with $k^{2}$ circles of diameter 1/k distributed in ${\left[0,1\right]}^{2}$ on a square grid.

### 4.6. A Remark on the Work of Amigó et al.

**Figure 3:**
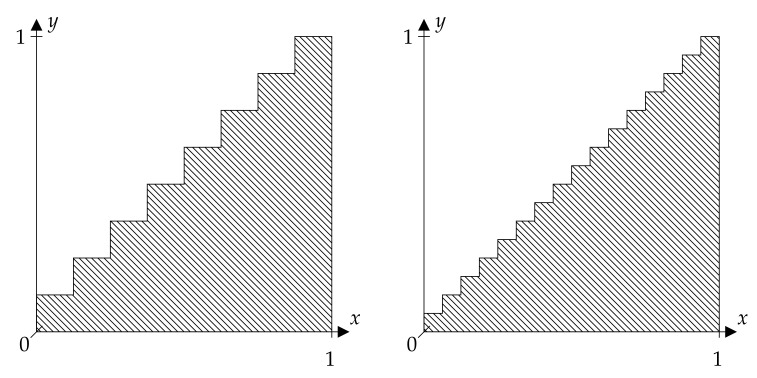
$R_{k}:=\left\{\left(x,y\right)\in R^{2}|\left\lfloor k\cdot x\right\rfloor \geq \left\lfloor k\cdot y\right\rfloor \right\}$ for k=8 (left side) and k=16 (right side), only shown in ${\left[0.1\right]}^{2}$.

Consider the discriminating relation:

$$R_{k}=\left\{\left(x,y\right)\in R^{2}|\left\lceil k\cdot x\right\rceil \geq \left\lceil k\cdot y\right\rceil \right\}$$

shown in Figure 3 for k∈N. Assume for simplicity that the dynamical system is defined on Ω=[0,1[ and that *X* is the identity map id. It is easy to see that:

$$\sigma \left(\overset{n}{\underset{t=0}{⋁}}T^{-t}\left(P_{k}\right)|n\in N\right){=}_{\mu }\sigma \left(f_{id}^{R_{k}}\circ T^{t}|t\in N_{0}\right)$$

holds true, where $P_{k}:=\{[\left(i-1\right)/k,i/k{\left[\right\}}_{i=1}^{k}$. Therefore, (9) in Theorem 1 holds true if $P_{k}$ is a generating partition.

Additionally, one could consider the quantity:

(18) $$\lim_{k\to \infty }\underset{n\to \infty }{lim inf}\frac{1}{n}H\left(\overset{n-1}{\underset{s=0}{⋁}}\overset{n-1}{\underset{t=s+1}{⋁}}{\left(T^{s},T^{t}\right)}^{-1}\left(\left\{R_{k},R^{2}\backslash R_{k}\right\}\right)\right)$$

which was introduced by Amigó et. al. [9]. They used finite-valued random variables to quantize the dynamical system into *k* parts and considered the ordinal patterns of the quantized systems while we directly apply the quantization to the discriminating relation. Both approaches only differ in their notation. They showed in their paper that the limit in (18) is equal to the Kolmogorov–Sinai entropy.

## 5. Proof of the Main Statement

We first recall some definitions and statements related to partitions and the conditional entropy. For two partitions P,Q⊂A of Ω, the *conditional entropy* is defined as

H(P|Q):=H(P∨Q)−H(Q).

Roughly speaking, the conditional entropy H(P|Q) describes how much uncertainty is left in the outcomes described by the sets given in P if one already has information about the outcomes described by the sets given in Q. For example, if P=Q, then H(P|Q)=0. However, if P and Q are independent, meaning that μ(P∩Q)=μ(P)·μ(Q) for all P∈P and Q∈Q, and H(P|Q)=H(Q).

Without explicitly referencing them, we will use the following properties of the conditional entropy:

(i) $H(T^{-1}\left(P\right)|T^{-1}\left(Q\right))=H\left(P|Q\right)$,
(ii) $H\left({⋁}_{i=1}^{n}P_{i}|Q\right)\leq \sum_{i=1}^{n}H\left(P_{i}|Q\right)$,
(iii) $H\left(P|Q_{1}\vee Q_{2}\right)\leq H\left(P|Q_{1}\right)$.

See, for examples, [7] for proofs.

A sequence of partitions ${\left(P_{i}\right)}_{i\in N}$ in A of Ω is said to be generating (the σ-algebra A), if $\sigma \left(P_{i}\right)\subseteq \sigma \left(P_{i+1}\right)$ for all i∈N and:

$$\sigma \left(P_{i}|i\in N\right){=}_{\mu }A$$

holds true. As a consequence of this property:

(19) $$\lim_{n\to \infty }H\left(P|P_{n}\right)=0$$

holds true for all partitions P⊂A of Ω. Using the properties of the conditional entropy implies:

$$h\left(T\right)=\lim_{n\to \infty }h\left(T,P_{n}\right).$$

For N∈N:

$$U_{N}=\left\{[\left(i-1\right)/2^{N},i/2^{N}[|i\in \left\{1,2,\ldots ,2^{N}-1\right\}\right\}\cup \left\{\left[\left(2^{N}-1\right)/2^{N},1\right]\right\}$$

will denote the partition of [0,1] in $2^{N}$ equally sized intervals.

We start the proof of Theorem 1 with two basic lemmata.

**Lemma** **3.** *Let (Ω,A,μ,T) be a measure-preserving dynamical system, $X=(X_{1},X_{2},\ldots ,X_{d})$ be a random vector and Y be a random variable satisfying (9). Then, there exists some constant c∈R with:*

$$h\left(T^{m}\right)\leq \lim_{N\to \infty }h\left(T^{m},\overset{d}{\underset{i=1}{⋁}}\overset{m-1}{\underset{t=0}{⋁}}T^{-t}\left(X_{i}^{-1}\left(f_{X_{i}}^{-1}\left(U_{N}\right)\right)\right)\right)+c.$$

*for all m∈N.*

**Proof.**  Fix m∈N. Set:

$$M:=\left\{Y^{-1}\left(y\right)|y\in Y\left(\Omega \right)\right\}$$

Since *Y* was assumed to attain only a finite number of different values, M is a finite partition of Ω. Because the Borel σ-algebra of [0,1] is generated by the partitions $U_{N}$ and due to (9), we have:

$$\begin{array}{cc} A & {=}_{\mu }\sigma \left(\left(f_{X_{i}}\circ X_{i}\circ T^{t},Y\right)|t\in N_{0},i\in \left\{1,2,\ldots ,d\right\}\right)\\ \; & =\sigma \left(T^{-t}\left(X_{i}^{-1}\left(f_{X_{i}}^{-1}\left(U_{N}\right)\right)\right)\vee M|N\in N,t\in N_{0},i\in \left\{1,2,\ldots ,d\right\}\right). \end{array}$$

Thus, for any ε>0 and any finite partition P⊂A of Ω, there exists an $N_{\varepsilon }\in N$ and a $t_{\varepsilon }\in N$ with:

$$\begin{array}{cc} h(T^{m},P) & \leq h\left(T^{m},\overset{d}{\underset{i=1}{⋁}}\overset{t_{\varepsilon }-1}{\underset{t=0}{⋁}}T^{-t}\left(X_{i}^{-1}\left(f_{X_{i}}^{-1}\left(U_{N}\right)\right)\right)\vee M\right)+\varepsilon \\ \; & \leq h\left(T^{m},\overset{d}{\underset{i=1}{⋁}}\overset{t_{\varepsilon }-1}{\underset{t=0}{⋁}}T^{-t}\left(X_{i}^{-1}\left(f_{X_{i}}^{-1}\left(U_{N}\right)\right)\right)\right)+h\left(T^{m},M\right)+\varepsilon \\ \; & \leq h\left(T^{m},\overset{d}{\underset{i=1}{⋁}}\overset{t_{\varepsilon }-1}{\underset{t=0}{⋁}}T^{-t}\left(X_{i}^{-1}\left(f_{X_{i}}^{-1}\left(U_{N}\right)\right)\right)\right)+H\left(M\right)+\varepsilon \\ \; & \leq h\left(T^{m},\overset{d}{\underset{i=1}{⋁}}\overset{m-1}{\underset{t=0}{⋁}}T^{-t}\left(X_{i}^{-1}\left(f_{X_{i}}^{-1}\left(U_{N}\right)\right)\right)\right)+H\left(M\right)+\varepsilon \end{array}$$

for all $N\geq N_{\varepsilon }$. Hence:

$$h\left(T^{m},P\right)\leq \lim_{N\to \infty }h\left(T^{m},\overset{d}{\underset{i=1}{⋁}}\overset{m-1}{\underset{t=0}{⋁}}T^{-t}\left(X_{i}^{-1}\left(f_{X_{i}}^{-1}\left(U_{N}\right)\right)\right)\right)+H\left(M\right)+\varepsilon$$

for any ε>0, which implies:

$$\begin{array}{cc} h(T^{m}) & =\underset{P}{\sup}h\left(T^{m},P\right)\\ \; & \leq \lim_{N\to \infty }h\left(T^{m},\overset{d}{\underset{i=1}{⋁}}\overset{m-1}{\underset{t=0}{⋁}}T^{-t}\left(X_{i}^{-1}\left(f_{X_{i}}^{-1}\left(U_{N}\right)\right)\right)\right)+H\left(M\right). \end{array}$$

□

**Lemma** **4.** *Let (Ω,A,μ) be a probability space, X:Ω→R be a random variable and $A,B\in B(R^{2})$. Then:*

$$\int \left|f_{X}^{A}-f_{X}^{B}\right|\;d\mu_{X}\leq \mu_{X}^{2}\left(A▵B\right)$$

*holds true.*

**Proof.**  For all x∈R:

$$\begin{array}{cc} \; & \;\mu (\{\omega \in \Omega |(x,X(\omega ))\in A\Delta B\})\\ = & \;\mu (\{\omega \in \Omega |(x,X(\omega ))\in A\backslash B\})+\mu (\{\omega \in \Omega |(x,X(\omega ))\in B\backslash A\})\\ \geq & \;\mu (\{\omega \in \Omega |(x,X(\omega ))\in A\backslash B\})\\ \geq & \;\mu (\{\omega \in \Omega |(x,X(\omega ))\in A\})-\mu (\{\omega \in \Omega |(x,X(\omega ))\in B\})\\ = & \;f_{X}^{A}\left(x\right)-f_{X}^{B}\left(x\right) \end{array}$$

holds true. Analogously, one can show:

$$\mu \left(\left\{\omega \in \Omega |\left(x,X\left(\omega \right)\right)\in A\Delta B\right\}\right)\geq f_{X}^{B}\left(x\right)-f_{X}^{A}\left(x\right).$$

This implies:

$$\mu \left(\left\{\omega \in \Omega |\left(x,X\left(\omega \right)\right)\in A\Delta B\right\}\right)\geq \left|f_{X}^{A}\left(x\right)-f_{X}^{B}\left(x\right)\right|$$

and, by Fubini’s theorem:

$$\begin{array}{cc} \; & \int \left|f_{X}^{A}-f_{X}^{B}\right|\;d\mu_{X}\left(x\right)\\ \; & \leq \int \mu \left(\left\{\omega \in \Omega |\left(x,X\left(\omega \right)\right)\in A\Delta B\right\}\right)\;d\mu_{X}\left(x\right)\\ \; & =\int \int 1_{\left\{\omega \in \Omega |(x,X(\omega ))\in A\Delta B\right\}}\left(\omega^{\prime }\right)\;d\mu \left(\omega^{\prime }\right)\;d\mu_{X}\left(x\right)\\ \; & =\int \int 1_{\left\{y\in R|(x,y)\in A\Delta B\right\}}\left(y^{\prime }\right)\;d\mu_{X}\left(y^{\prime }\right)\;d\mu_{X}\left(x\right)\\ \; & =\int_{R^{2}}1_{A\Delta B}\left(x,y^{\prime }\right)\;d\mu_{X}^{2}\left(x,y^{\prime }\right)\\ \; & =\mu_{X}^{2}\left(A\Delta B\right). \end{array}$$

□

Therefore, in particular, the above lemma implies that, if ${\left(R_{j}\right)}_{j\in N}$ is a sequence of sets in $B(R^{2})$ with $\lim_{j\to \infty }\mu_{X}^{2}\left(R_{j}▵R\right)=0$, then $f_{X}^{R_{j}}$ converges to $f_{X}^{R}$ in $L^{1}$ for j→∞.

Given $R\subseteq R^{2}$ and a random variable X:Ω→R, consider the function $f_{X,n}^{R}:\Omega \times R\to \left[0,1\right]$ with:

$$f_{X,n}^{R}\left(x,\omega \right):=\frac{1}{n}\#\left\{t\in \left\{1,2,\ldots ,n\right\}|\left(x,X\left(T^{t}\left(\omega \right)\right)\right)\in R\right\}.$$

We want to show that $f_{X,n}^{R}\left(x,\omega \right)$ converges to $f_{X}^{R}\left(x\right)$ for all x∈R and μ-almost all ω∈Ω. If $f_{X,n}^{R}\left(x,\omega \right)$ is monotone in *x* for all ω∈Ω and n∈N, this can be shown relatively easily using the pointwise ergodic theorem and the monotonicity of the considered functions. Monotonicity is guaranteed, if $x_{1}\leq x_{2}$ implies:

$$\left\{y\in R|\left(x_{1},y\right)\in R\right\}\subseteq \left\{y\in R|\left(x_{2},y\right)\in R\right\}.$$

For example, if $R=\{\left(x,y\right)\in R^{2}|x>y\}$, the above implication holds true. For this special case, a proof of the statement in Lemma 5 can be found in [4].

However, we are interested in general sets $R\in B(R^{2})$ and therefore, cannot use the monotonicity. Therefore, we have to prove this statement differently.

**Lemma** **5.** *Let (Ω,A,μ,T) be an ergodic measure-preserving dynamical system,* *$X=(X_{1},X_{2},\ldots ,X_{d})$ be a random vector and $R\in B(R^{2})$ satisfy (8). Then, for all i∈{1,2,…,d}, there exist sets $\tilde{\Omega }\in A$ and B∈B(R) with $\mu \left(\tilde{\Omega }\right)=\mu_{X_{i}}\left(B\right)=1$ satisfying:*

$$\lim_{n\to \infty }f_{X_{i},n}^{R}\left(x,\omega \right)=f_{X_{i}}^{R}\left(x\right)$$

*for all $\omega \in \tilde{\Omega }$ and x∈B.*

**Proof.**  Fix i∈{1,2,…,d}. According to (8), there exists a countable set

$C=\{\left(a_{k},b_{k}\right)|k\in N\}$ with:

$$\mu_{X_{i}}^{2}\left(\partial R\backslash C\right)=0.$$

By the pointwise ergodic theorem (see, e.g., [10]), for all j,k∈N, there exists $\Omega_{j,k}^{*}\in A$ with $\mu (\Omega_{j,k}^{*})=1$ and:

$$\lim_{n\to \infty }f_{X_{i},n}^{\left\{\left(a_{k},b_{k}\right)\right\}}\left(a_{j},\omega \right)=f_{X_{i}}^{\left\{\left(a_{k},b_{k}\right)\right\}}\left(a_{j}\right)$$

for all $\omega \in \Omega_{j,k}^{*}$. It is easy to see that:

$$f_{X_{i},n}^{\left\{\left(a_{k},b_{k}\right)\right\}}\left(x,\omega \right)=0=f_{X_{i}}^{\left\{\left(a_{k},b_{k}\right)\right\}}\left(x\right)$$

holds true for all n∈N and ω∈Ω if $x\neq a_{k}$. Hence:

(20) $$\lim_{n\to \infty }f_{X_{i},n}^{\left\{\left(a_{k},b_{k}\right)\right\}}\left(x,\omega \right)=f_{X_{i}}^{\left\{\left(a_{k},b_{k}\right)\right\}}\left(x\right)$$

for all x∈R and $\omega \in {⋂}_{j=1}^{\infty }\Omega_{j,k}^{*}$. Using Fatou’s lemma and the fact that *C* is countable implies:

(21) $$\begin{array}{cc} \underset{n\to \infty }{lim inf}f_{X_{i},n}^{R\cap C}\left(x,\omega \right) & =\underset{n\to \infty }{lim inf}\sum_{\begin{array}{c} k\in N:\\ (a_{k},b_{k})\in R \end{array}}f_{X_{i},n}^{\left\{\left(a_{k},b_{k}\right)\right\}}\left(x,\omega \right)\\ & \geq \sum_{\begin{array}{c} k\in N:\\ (a_{k},b_{k})\in R \end{array}}\underset{n\to \infty }{lim inf}f_{X_{i},n}^{\left\{\left(a_{k},b_{k}\right)\right\}}\left(x,\omega \right)\\ \; & \overset{\left(20\right)}{=}\sum_{\begin{array}{c} k\in N:\\ (a_{k},b_{k})\in R \end{array}}f_{X_{i}}^{\left\{\left(a_{k},b_{k}\right)\right\}}\left(x\right)=f_{X_{i}}^{R\cap C}\left(x\right) \end{array}$$

for all x∈R and $\omega \in {⋂}_{j=1}^{\infty }{⋂}_{k=1}^{\infty }\Omega_{j,k}^{*}$. We will use this fact later.

Since R\∂R is open, there exists a countable collection of pairwise disjoint rectangles $A_{j}\subseteq R^{2}$ with:

(22) $$R\backslash \partial R=\overset{\infty }{\underset{j=1}{⋃}}A_{j}.$$

Take $\left(x_{j},y_{j}\right)\in A_{j}$ for all j∈N. Using the pointwise ergodic theorem, for all j∈N there exists a set $\Omega_{j}\in A$ with $\mu (\Omega_{j})=1$ and:

(23) $$\begin{array}{c} \lim_{n\to \infty }f_{X_{i},n}^{A_{j}}\left(x_{j},\omega \right)=f_{X_{i}}^{A_{j}}\left(x_{j}\right) \end{array}$$

for all $\omega \in \Omega_{j}$. Because $A_{j}$ is a rectangle, for all ω∈Ω:

$$\begin{array}{cc} f_{X_{i},n}^{A_{j}}\left(x,\omega \right) & =f_{X_{i},n}^{A_{j}}\left(x_{j},\omega \right)\\ \mathrm{and}\;f_{X_{i}}^{A_{j}}\left(x\right) & =f_{X_{i}}^{A_{j}}\left(x_{j}\right) \end{array}$$

holds true for all x∈R with $\left\{x\right\}\times R\cap A_{i}\neq \emptyset$ and:

$$\begin{array}{c} f_{X_{i},n}^{A_{j}}\left(x,\omega \right)=f_{X_{i}}^{A_{j}}\left(x\right)=0 \end{array}$$

holds true for all x∈R with $R\times \left\{x\right\}\cap A_{i}=\emptyset$. Together with (23), this implies:

(24) $$\lim_{n\to \infty }f_{X_{i},n}^{A_{j}\backslash C}\left(x,\omega \right)=f_{X_{i}}^{A_{j}\backslash C}\left(x\right)$$

for all x∈R and $\omega \in \Omega_{j}$.

Set $R_{J}:={⋃}_{j=1}^{J}A_{j}$. Lemma 4 provides:

$$\begin{array}{cc} \; & \lim_{J\to \infty }\int \left|f_{X_{i}}^{R_{J}\backslash C}\left(x\right)-f_{X_{i}}^{R\backslash C}\left(x\right)\right|\;d\mu_{X}\left(x\right)\\ \; & =\lim_{J\to \infty }\mu_{X_{i}}^{2}\left(\left(R_{J}\backslash C\right)▵\left(R\backslash C\right)\right)\\ \; & =\lim_{J\to \infty }\mu_{X_{i}}^{2}\left(\left(R\backslash C\right)\backslash \left(R_{J}\backslash C\right)\right)\\ \; & =\lim_{J\to \infty }\mu_{X_{i}}^{2}\left(R\backslash \left(R_{J}\cup C\right)\right)\\ \; & =\lim_{J\to \infty }\mu_{X_{i}}^{2}\left(\left(\left(R\cap \partial R\right)\cup \left(R\backslash \partial R\right)\right)\backslash \left(R_{J}\cup C\right)\right)\\ \; & =\lim_{J\to \infty }\mu_{X_{i}}^{2}\left(\left(R\cap \partial R\right)\backslash \left(R_{J}\cup C\right)\right)+\mu_{X_{i}}^{2}\left(\left(R\backslash \partial R\right)\backslash \left(R_{J}\cup C\right)\right)\\ \; & \leq \lim_{J\to \infty }\mu_{X_{i}}^{2}\left(\partial R\backslash C\right)+\mu_{X_{i}}^{2}\left(\left(R\backslash \partial R\right)\backslash R_{J}\right)\\ \; & \overset{\left(8\right)}{=}\lim_{J\to \infty }\mu_{X_{i}}^{2}\left(\left(R\backslash \partial R\right)\backslash R_{J}\right)\\ \; & \overset{\left(22\right)}{=}\mu_{X_{i}}^{2}\left(\left(R\backslash \partial R\right)\backslash \left(R\backslash \partial R\right)\right)=0. \end{array}$$

Therefore, there exists a set $B_{1}$ with $\mu_{X_{i}}\left(B_{1}\right)=1$ and a sequence ${\left(J_{k}\right)}_{k\in N}$ with:

(25) $$\lim_{k\to \infty }f_{X_{i}}^{R_{J_{k}}\backslash C}\left(x\right)=f_{X_{i}}^{R\backslash C}\left(x\right)$$

for all $x\in B_{1}$. Thus:

$$\begin{array}{cc} \underset{n\to \infty }{lim inf}f_{X_{i},n}^{R}\left(x,\omega \right) & \geq \underset{n\to \infty }{lim inf}f_{X_{i},n}^{R\cap C}\left(x,\omega \right)+\underset{n\to \infty }{lim inf}f_{X_{i},n}^{R\backslash C}\left(x,\omega \right)\\ \; & \overset{\left(21\right)}{\geq }f_{X_{i}}^{R\cap C}\left(x\right)+\underset{n\to \infty }{lim inf}f_{X_{i},n}^{R_{J_{k}}\backslash C}\left(x,\omega \right)\\ \; & \geq f_{X_{i}}^{R\cap C}\left(x\right)+\lim_{k\to \infty }\underset{n\to \infty }{lim inf}f_{X_{i},n}^{R_{J_{k}}\backslash C}\left(x,\omega \right)\\ \; & =f_{X_{i}}^{R\cap C}\left(x\right)+\lim_{k\to \infty }\underset{n\to \infty }{lim inf}\sum_{j=1}^{J_{k}}f_{X_{i},n}^{A_{j}\backslash C}\left(x,\omega \right)\\ \; & \geq f_{X_{i}}^{R\cap C}\left(x\right)+\lim_{k\to \infty }\sum_{j=1}^{J_{k}}\underset{n\to \infty }{lim inf}f_{X_{i},n}^{A_{j}\backslash C}\left(x,\omega \right)\\ \; & \overset{\left(24\right)}{=}f_{X_{i}}^{R\cap C}\left(x\right)+\lim_{k\to \infty }\sum_{j=1}^{J_{k}}f_{X_{i}}^{A_{j}\backslash C}\left(x\right)\\ \; & =f_{X_{i}}^{R\cap C}\left(x\right)+\lim_{k\to \infty }f_{X_{i}}^{R_{J_{k}}\backslash C}\left(x\right)\\ \; & \overset{\left(25\right)}{=}f_{X_{i}}^{R\cap C}\left(x\right)+f_{X_{i}}^{R\backslash C}\left(x\right)\\ \; & =f_{X_{i}}^{R}\left(x\right) \end{array}$$

for all $x\in B_{1}$ and $\omega \in {\tilde{\Omega }}_{1}:={⋂}_{j=1}^{\infty }{⋂}_{k=1}^{\infty }\Omega_{j}\cap \Omega_{j,k}^{*}$. Because $R^{c}\backslash \partial R$ is open as well, one can analogously show that there exist sets ${\tilde{\Omega }}_{2}\in A$ and $B_{2}\in B\left(R\right)$ with $\mu \left({\tilde{\Omega }}_{2}\right)=\mu_{X_{i}}\left(B_{2}\right)=1$ and:

$$\underset{n\to \infty }{lim inf}f_{X_{i},n}^{R^{c}}\left(x,\omega \right)\geq f_{X_{i}}^{R^{c}}\left(x\right)$$

for all $x\in B_{2}$ and $\omega \in {\tilde{\Omega }}_{2}$. This implies:

$$\begin{array}{c} \underset{n\to \infty }{lim sup}f_{X_{i},n}^{R}\left(x,\omega \right)=1-\underset{n\to \infty }{lim inf}f_{X_{i},n}^{R^{c}}\left(x,\omega \right)\leq 1-f_{X_{i}}^{R^{c}}\left(x\right)=f_{X_{i}}^{R}\left(x\right). \end{array}$$

Hence:

(26) $$f_{X_{i}}^{R}\left(x\right)\leq \underset{n\to \infty }{lim inf}f_{X_{i},n}^{R}\left(x,\omega \right)\leq \underset{n\to \infty }{lim sup}f_{X_{i},n}^{R}\left(x,\omega \right)\leq f_{X_{i}}^{R}\left(x\right)$$

for all $x\in B:=B_{1}\cap B_{2}$ and $\omega \in \tilde{\Omega }:={\tilde{\Omega }}_{1}\cap {\tilde{\Omega }}_{2}$. □

Given a random vector $\left(X_{1},X_{2},\ldots ,X_{d}\right)$ and $R\in B(R^{2})$, we define the partition:

$$R_{i}:={\left(X_{i},X_{i}\right)}^{-1}\left(\left\{R,R^{c}\right\}\right)$$

for all i∈{1,2,…,d}, which is equal to:

$$\begin{array}{cc} R_{i}=\{ & \{\left(\omega_{1},\omega_{2}\right)\in \Omega^{2}|\left(X_{i}\left(\omega_{1}\right),X_{i}\left(\omega_{2}\right)\right)\in R\},\\ \; & \{\left(\omega_{1},\omega_{2}\right)\in \Omega^{2}|\left(X_{i}\left(\omega_{1}\right),X_{i}\left(\omega_{2}\right)\right)\notin R\}\}. \end{array}$$

**Lemma** **1.** *Let (Ω,A,μ,T) be an ergodic measure-preserving dynamical system,* *$X=\left(X_{1},X_{2},\ldots ,X_{d}\right):\Omega \to R^{d}$ be a random vector, ${\left(E_{n}\right)}_{n\in N}$ be a timing and $R\in B(R^{2})$ satisfying (8). Then, there exists a sequence ${\left(n_{k}\right)}_{k\in N}\subseteq N_{0}^{N}$ with:*

$$\lim_{k\to \infty }H\left(T^{-n_{k}}\left({\left(f_{X_{i}}\circ X_{i}\right)}^{-1}\left(U_{N}\right)\right)\left|\overset{n_{k}}{\underset{v=0}{⋁}}T^{-v}\left(\underset{\left(s,t\right)\in E_{k}}{⋁}{\left(T^{s},T^{t}\right)}^{-1}\left(R_{i}\right)\right)\right.\right)=0$$

*for all N∈N and i∈{1,2,…,d}.*

**Proof.**  Because ${\left(E_{n}\right)}_{n\in N}$ is a timing, there exist a sequence ${\left(a_{n}\right)}_{n\in N}\in N_{0}^{N}$ with:

$$\underset{N\to \infty }{lim sup}\frac{\#\left\{n\in \left\{1,2,\ldots ,N\right\}|\left(a_{n},a_{n}+n\right)\in {⋃}_{k=1}^{\infty }E_{k}\right\}}{N}=1.$$

So one can find a strictly increasing sequence ${\left(N_{n}\right)}_{n\in N}\in N^{N}$ with:

$$\left(a_{N_{n}},a_{N_{n}}+N_{n}\right)\in \overset{\infty }{\underset{k=1}{⋃}}E_{k}$$

for all n∈N and:

(27) $$\underset{n\to \infty }{lim sup}\frac{n}{N_{n}}=1.$$

Now, fix i∈{1,2,…,d}. According to Lemma 5, there exist sets $\tilde{\Omega }\in A$ and B∈B(R) with $\mu \left(\tilde{\Omega }\right)=\mu_{X_{i}}\left(B\right)=1$ satisfying:

$$\lim_{n\to \infty }f_{X_{i},n}^{R}\left(\omega ,x\right)=f_{X_{i}}^{R}\left(x\right)$$

for all $\omega \in \tilde{\Omega }$ and x∈B. Set

$$\Omega_{0}:=\tilde{\Omega }\cap X_{i}^{-1}\left(B\right).$$

Consider the function $\phi_{n}:\Omega \to \left[0,1\right]$ with:

$$\phi_{n}\left(\omega \right):=\frac{1}{N_{n}}\#\left\{t\in \left\{N_{1},N_{2},\ldots ,N_{n}\right\}|\left(X_{i}\left(T^{t}\left(\omega \right)\right),X_{i}\left(\omega \right)\right)\in R\right\}.$$

Then:

(28) $$\begin{array}{cc} \; & \;f_{X_{i}}^{R}\left(X_{i}\left(\omega \right)\right)\\ = & \;\lim_{n\to \infty }f_{X_{i},n}^{R}\left(\omega ,X_{i}\left(\omega \right)\right)\\ = & \;\underset{n\to \infty }{lim sup}f_{X_{i},N_{n}}^{R}\left(\omega ,X_{i}\left(\omega \right)\right)\\ \geq & \;\underset{n\to \infty }{lim sup}\phi_{n}\left(\omega \right)\\ = & \;\underset{n\to \infty }{lim sup}\left[f_{X_{i},N_{n}}^{R}\left(\omega ,X_{i}\left(\omega \right)\right)\right.\\ \; & -\left.\frac{1}{N_{n}}\#\left\{t\in \left\{1,2,\ldots ,N_{n}\right\}\backslash \left\{N_{1},N_{2},\ldots ,N_{n}\right\}|\left(X_{i}\left(T^{t}\left(\omega \right)\right),X_{i}\left(\omega \right)\right)\in R\right\}\right]\\ \geq & \;\underset{n\to \infty }{lim sup}\left[f_{X_{i},N_{n}}^{R}\left(\omega ,X_{i}\left(\omega \right)\right)-\frac{1}{N_{n}}\#\left\{1,2,\ldots ,N_{n}\right\}\backslash \left\{N_{1},N_{2},\ldots ,N_{n}\right\}\right]\\ = & \;\underset{n\to \infty }{lim sup}\left[f_{X_{i},N_{n}}^{R}\left(\omega ,X_{i}\left(\omega \right)\right)-\frac{N_{n}-n}{N_{n}}\right]\\ = & \;\lim_{n\to \infty }f_{X_{i},N_{n}}^{R}\left(\omega ,X_{i}\left(\omega \right)\right)-1+\underset{n\to \infty }{lim sup}\frac{n}{N_{n}}\\ \overset{\left(27\right)}{=} & \;f_{X_{i}}^{R}\left(X_{i}\left(\omega \right)\right) \end{array}$$

for all $\omega \in \Omega_{0}$.

It is easy to see that:

$$\sigma \left(\phi_{n}\right)\subseteq \sigma \left(\overset{k}{\underset{t=1}{⋁}}{\left(id,T^{N_{t}}\right)}^{-1}\left(R_{i}\right)|k\in N\right)$$

holds true for all n∈N. This implies (see for instance [11], Theorem 13.4 (i)):

$$\sigma \left(f_{X_{i}}^{R}\circ X_{i}\right)\overset{\left(28\right)}{{=}_{\mu }}\sigma \left(\underset{n\to \infty }{lim sup}\phi_{n}\right)\subseteq \sigma \left(\overset{k}{\underset{t=1}{⋁}}{\left(id,T^{N_{t}}\right)}^{-1}\left(R_{i}\right)|k\in N\right).$$

Therefore ${⋁}_{t=1}^{k}{\left(id,T^{N_{t}}\right)}^{-1}\left(R_{i}\right)$ is a sequence of partitions generating $\sigma \left(f_{X_{i}}^{R}\circ X_{i}\right)$. By (19), this implies:

(29) $$\lim_{k\to \infty }H\left({\left(f_{X_{i}}\circ X_{i}\right)}^{-1}\left(U_{N}\right)\left|\overset{k}{\underset{t=1}{⋁}}{\left(id,T^{N_{t}}\right)}^{-1}\left(R_{i}\right)\right.\right)=0$$

for all N∈N. Set:

$$n_{k}:=\max_{1\leq n\leq k}a_{N_{n}}.$$

Notice that:

$$\sigma \left(T^{-n_{k}}\left(\overset{k}{\underset{t=1}{⋁}}{\left(id,T^{N_{t}}\right)}^{-1}\left(R_{i}\right)\right)\right)\subseteq \sigma \left(\overset{n_{k}}{\underset{v=0}{⋁}}T^{-v}\left(\underset{\left(s,t\right)\in E_{k}}{⋁}{\left(T^{s},T^{t}\right)}^{-1}\left(R_{i}\right)\right)\right)$$

holds true for all k∈N. Consequently:

$$\begin{array}{cc} \; & \lim_{k\to \infty }H\left(T^{-n_{k}}\left({\left(f_{X_{i}}\circ X_{i}\right)}^{-1}\left(U_{N}\right)\right)\left|\overset{n_{k}}{\underset{v=0}{⋁}}T^{-v}\left(\underset{\left(s,t\right)\in E_{k}}{⋁}{\left(T^{s},T^{t}\right)}^{-1}\left(R_{i}\right)\right)\right.\right)\\ \; & \leq \lim_{k\to \infty }H\left(T^{-n_{k}}\left({\left(f_{X_{i}}\circ X_{i}\right)}^{-1}\left(U_{N}\right)\right)\left|T^{-n_{k}}\left(\overset{k}{\underset{t=1}{⋁}}{\left(id,T^{N_{t}}\right)}^{-1}\left(R_{i}\right)\right)\right.\right)\\ \; & =\lim_{k\to \infty }H\left({\left(f_{X_{i}}\circ X_{i}\right)}^{-1}\left(U_{N}\right)\left|\overset{k}{\underset{t=1}{⋁}}{\left(id,T^{N_{t}}\right)}^{-1}\left(R_{i}\right)\right.\right)\overset{\left(29\right)}{=}0 \end{array}$$

for all N∈N. □

We can now finalize the proof of Theorem 1.

**Proof of Theorem 1**. Let N∈N and m∈N. Set:

$$P_{N}^{i}:=X_{i}^{-1}\left(f_{X_{i}}^{-1}\left(U_{N}\right)\right)$$

and:

$$Q_{k}^{i}:=\underset{\left(s,t\right)\in E_{k}}{⋁}{\left(T^{s},T^{t}\right)}^{-1}\left(R_{i}\right)$$

for all i∈{1,2,…,d} and k∈N. According to Lemma 1, there exists a sequence ${\left(n_{k}\right)}_{k\in N}\subseteq N_{0}^{N}$ with:

(30) $$\lim_{k\to \infty }H\left(T^{-n_{k}}\left(P_{N}^{i}\right)\left|\overset{n_{k}}{\underset{v=0}{⋁}}T^{-v}\left(Q_{k}^{i}\right)\right.\right)=0$$

for all i∈{1,2,…,d}. We have:

$$\begin{array}{cc} \; & \lim_{n\to \infty }\frac{1}{n}H\left(\overset{d}{\underset{i=1}{⋁}}\overset{nm-1}{\underset{u=0}{⋁}}T^{-u}\left(P_{N}^{i}\right)\left|\overset{d}{\underset{i=1}{⋁}}\overset{nm-1}{\underset{u=0}{⋁}}T^{-u}\left(Q_{k}^{i}\right)\right.\right)\\ \leq & \sum_{i=1}^{d}\lim_{n\to \infty }\frac{1}{n}H\left(\overset{nm-1}{\underset{u=0}{⋁}}T^{-u}\left(P_{N}^{i}\right)\left|\overset{nm-1}{\underset{u=0}{⋁}}T^{-u}\left(Q_{k}^{i}\right)\right.\right)\\ = & \sum_{i=1}^{d}\lim_{n\to \infty }\frac{1}{n}H\left(\overset{nm-1-n_{k}}{\underset{u=0}{⋁}}T^{-u}\left(T^{-n_{k}}\left(P_{N}^{i}\right)\right)\left|\overset{nm-1-n_{k}}{\underset{u=0}{⋁}}T^{-u}\left(\overset{n_{k}}{\underset{v=0}{⋁}}T^{-v}\left(Q_{k}^{i}\right)\right)\right.\right)\\ \leq & \sum_{i=1}^{d}\lim_{n\to \infty }\frac{1}{n}\sum_{u=0}^{nm-1-n_{k}}H\left(T^{-u}\left(T^{-n_{k}}\left(P_{N}^{i}\right)\right)\left|\overset{n_{k}}{\underset{v=0}{⋁}}T^{-v}\left(Q_{k}^{i}\right)\right.\right)\\ = & \sum_{i=1}^{d}\lim_{n\to \infty }\frac{nm-1-n_{k}}{n}H\left(T^{-n_{k}}\left(P_{N}^{i}\right)\left|\overset{n_{k}}{\underset{v=0}{⋁}}T^{-v}\left(Q_{k}^{i}\right)\right.\right)\\ \leq & \sum_{i=1}^{d}m\cdot H\left(T^{-n_{k}}\left(P_{N}^{i}\right)\left|\overset{n_{k}}{\underset{v=0}{⋁}}T^{-v}\left(Q_{k}^{i}\right)\right.\right) \end{array}$$

for all k,m,N∈N. This implies:

$$\begin{array}{cc} \; & h\left(T^{m},\overset{d}{\underset{i=1}{⋁}}\overset{m-1}{\underset{u=0}{⋁}}T^{-u}\left(P_{N}^{i}\right)\right)-\lim_{k\to \infty }h\left(T^{m},\overset{d}{\underset{i=1}{⋁}}\overset{m-1}{\underset{u=0}{⋁}}T^{-u}\left(Q_{k}^{i}\right)\right)\\ \; & \leq \lim_{k\to \infty }\lim_{n\to \infty }\frac{1}{n}H\left(\overset{d}{\underset{i=1}{⋁}}\overset{nm-1}{\underset{u=0}{⋁}}T^{-u}\left(P_{N}^{i}\right)\left|\overset{d}{\underset{i=1}{⋁}}\overset{nm-1}{\underset{u=0}{⋁}}T^{-u}\left(Q_{k}^{i}\right)\right.\right)\\ \; & \leq \lim_{k\to \infty }\sum_{i=1}^{d}m\cdot H\left(T^{-n_{k}}\left(P_{N}^{i}\right)\left|\overset{n_{k}}{\underset{v=0}{⋁}}T^{-v}\left(Q_{k}^{i}\right)\right.\right)\overset{\left(30\right)}{=}0. \end{array}$$

Using Lemma 3, we can conclude that there exists a constant c∈R with:

$$\begin{array}{cc} h(T^{m}) & \leq \lim_{k\to \infty }h\left(T^{m},\overset{d}{\underset{i=1}{⋁}}\overset{m-1}{\underset{u=0}{⋁}}T^{-u}\left(\underset{\left(s,t\right)\in E_{k}}{⋁}{\left(T^{s},T^{t}\right)}^{-1}\left(R_{i}\right)\right)\right)+c\\ \; & =\lim_{k\to \infty }m\cdot h\left(T,\overset{d}{\underset{i=1}{⋁}}\underset{\left(s,t\right)\in E_{k}}{⋁}{\left(T^{s},T^{t}\right)}^{-1}\left(R_{i}\right)\right)+c \end{array}$$

for all m∈N. Thus:

$$\begin{array}{cc} \; & \;h\left(T\right)-\lim_{k\to \infty }h\left(T,\overset{d}{\underset{i=1}{⋁}}\underset{\left(s,t\right)\in E_{k}}{⋁}{\left(T^{s},T^{t}\right)}^{-1}\left(R_{i}\right)\right)\\ \; & =\;\lim_{m\to \infty }\frac{1}{m}\cdot h\left(T^{m}\right)-\lim_{k\to \infty }h\left(T,\overset{d}{\underset{i=1}{⋁}}\underset{\left(s,t\right)\in E_{k}}{⋁}{\left(T^{s},T^{t}\right)}^{-1}\left(R_{i}\right)\right)\\ \; & \leq \lim_{m\to \infty }\frac{1}{m}\cdot c=0, \end{array}$$

which is equivalent to:

(31) $$h\left(T\right)\leq \lim_{k\to \infty }h\left(T,\overset{d}{\underset{i=1}{⋁}}\underset{\left(s,t\right)\in E_{k}}{⋁}{\left(T^{s},T^{t}\right)}^{-1}\left(R_{i}\right)\right).$$

On the other hand:

$$\begin{array}{c} h\left(T\right)=\underset{P}{\sup}h\left(T,P\right)\geq \lim_{k\to \infty }h\left(T,\overset{d}{\underset{i=1}{⋁}}\underset{\left(s,t\right)\in E_{k}}{⋁}{\left(T^{s},T^{t}\right)}^{-1}\left(R_{i}\right)\right), \end{array}$$

which, together with (31), finishes the proof. □

## 6. Conclusions

We discussed a special “two-dimensional” approach to symbolic dynamics differing from many usual approaches which was introduced in [6]. From the practical viewpoint, the difference can be illustrated as follows: given the time-dependent measurements of a real-valued quantity, a symbolization is not conducted for the measurements themselves as in usual approaches, but for pairs of measurements at two different times. This means that to each pair of possible measured values, a symbol from a finite symbol set is assigned. Here, we only considered two symbols which lead to a partitioning of the two-dimensional real space $R^{2}$ into a set *R* and its complement $R^{2}\backslash R$. In usual approaches, partitions of R are considered. (Advantages of the “two-dimensional” approach are described in [6]).

The set *R*, called a discriminating relation, was considered as a basic building block for constructing partitions of the state space of a given dynamical system, having time-dependent measurements of finitely many quantities in mind. In addition to the discrimination relation, the second central concept was the concept of a timing which roughly describes which pairs of times are included in the symbolization process and guarantees that there are not too few such pairs. The central question of the paper was that of under which conditions on a discriminating relation *R* the partitions constructed from *R* determine the KS entropy of a measure-preserving dynamical system. With Theorem 1, we gave a relatively general statement partially answering this question. Some specifications of the theorem in Section 4 illustrate the nature of “successful” discriminating relations.

Although the statement of Theorem 1 appears relatively natural when looking at the proofs a little closer, we do not expect that all cases where the K-S entropy can be constructed based on a discriminating relation is covered by the statement; however, we have no counterexample. The main tool used in the proofs of the results is the pointwise ergodic theorem. It allows to establish a connection between the generalized ordinal patterns and the shape of the discriminating relation.

The results of this paper, being on a rather abstract level, give some insights as to why the idea of ordinal patterns is working well, as reported by several applied papers, with extracting those advantageous features being more general than in the original ordinal approach. Having many choices for a discriminating relation, for practical purposes such as, for example, in a classification context, one needs methods and criteria for finding good discrimination relations, adapted to given data and problems. This is an important challenge for further research related to the given approach to symbolic dynamics. A further aspect is to discuss the approach for partitioning the $R^{2}$ into more than two pieces.
